# High-Temperature Oxidation Behavior of a Cu-Bearing 17Cr Ferritic Stainless Steel

**DOI:** 10.1155/2020/8847831

**Published:** 2020-12-16

**Authors:** Mengqi Zhang, Ying Han, Guoqing Zu, Jiapeng Sun, Weiwei Zhu, Hua Chen, Xu Ran

**Affiliations:** ^1^Key Laboratory of Advanced Structural Materials, Ministry of Education, Changchun University of Technology, Changchun 130012, China; ^2^College of Mechanics and Materials, Hohai University, Nanjing 211100, China

## Abstract

The isothermal oxidation behavior of 17Cr-0.85Si-0.5Nb-1.2Cu ferritic stainless steel in air was studied from 850°C to 1050°C by analyzing its weight gain after oxidation. The kinetic curves were plotted using the oxidation weight-gain data, and the structure, surface morphology, and element distribution of the oxide films were analyzed by XRD, SEM, and EDS. The results showed that the oxidation kinetics curves at 850°C and 950°C followed a parabolic law, and a continuous and dense oxide film composed of Cr_2_O_3_ and MnCr_2_O_4_, FeCr_2_O_4_, and Cu-Cr rich spinel was formed, which reveals that the steel displayed good oxidation resistance. When the temperature was increased to 1050°C, the oxidation kinetics curves gradually changed from parabolic to linear after 40 h exposure, which indicated that the oxidation resistance significantly worsened. A lower oxidation resistance was observed at 1050°C due to the formation of a large amount of Fe_2_O_3_ on the surface and the volatilization of the inner Cr_2_O_3_ layer.

## 1. Introduction

The materials used in automotive exhaust systems require high strength, good oxidation resistance, and satisfactory corrosion resistance because they are working in complex environments where includes cyclic oxidation and condensate corrosion [1–4]. Austenitic stainless steels are widely used to manufacture the hot-end parts of automobile exhaust systems, but in recent years, they have been gradually replaced by ferritic stainless steels due to their lower cost, smaller thermal expansion, and better high-temperature strength. Ferritic stainless steels have become one of the most promising candidates for use in exhaust manifolds [5–8].

As a relatively cheap alloying element, Cu added to Nb-bearing ferritic stainless steels are expected to greatly improve both strength and corrosion resistance; thus, Cu-Nb alloyed ferritic stainless steels have received great attention as a potential material for the hot-end parts of automotive exhaust systems [9–13]. For example, Kobayashi et al. [9, 10] investigated the effect of Nb addition on the aging precipitation behavior of 18%Cr-1.5%Cu ferritic stainless steel and proved that solute Nb atoms delayed the formation of Cu-rich regions and the coarsening of Cu particles in the steel. Ota et al. [11] studied the thermal fatigue properties of 15%-21% Cr ferritic stainless steel with various Cu and Nb contents. Guo et al. [12] reported that the combination of Cu and Nb improved the corrosion resistance of ferritic stainless steel in sulfuric acid solution.

High-temperature oxidation resistance is one of the important indicators for evaluating the performance of automotive exhaust pipe materials. Moreover, improving the oxidation property is significant to expand the application range for ferritic stainless steels after all. Sun et al. [14] studied the high-temperature cyclic oxidation behavior of 1Cr17 ferritic stainless steel in air and found the oxidation kinetic curves followed a parabolic law. Wei et al. [2] reported the influence of W and Ce on the oxidation behavior of ferritic stainless steel at 950-1100°C and indicated that the addition of W and Ce improved the density and adhesion of the oxide film, which increased the oxidation resistance. However, when the amount of W exceeded 1.0 wt.%, exfoliation of the oxide film was accelerated, leading to an increased oxidation rate. In addition, Chen et al. [15] investigated the oxidation of 441 ferritic stainless steel at 900-1050°C under a simulating automobile exhaust condition, and the results showed that the maximum service temperature is about 900°C, and the oxide film displayed a stratified structure comprised of a Mn-Cr spinel layer and a Cr_2_O_3_ layer. However, studies focusing on the oxidation mechanism of Cu-Nb alloyed ferritic stainless steel at high temperatures have not been reported yet. Thus, in this work, the high-temperature oxidation behavior of 17Cr-0.85Si-0.5Nb-1.2Cu ferritic stainless steel, a candidate material for automotive exhaust manifolds, was studied at 850°C, 950°C, and 1050°C in air, and the corresponding oxidation mechanism was proposed.

## 2. Materials and Methods

The material used in this work was 17Cr-0.85Si-0.5Nb-1.2Cu ferritic stainless steel, and the chemical composition is shown in Table 1. The steel was first hot-rolled to 10 mm and then annealed at 1100°C for 30 min, followed by water cooling. After that, specimens with sizes of 20 mm × 15 mm × 1.5 mm for the oxidation experiments were polished with SiC water-abrasive papers up to 1200 grit, washed with ethanol, and dried in a vacuum drying oven at 150°C.

Specimens that calcined to a constant weight were placed in the alumina crucibles, respectively, and stood against the inner wall of the crucible at a certain angle to ensure complete contact with air during the experiment. A box-type resistance heating furnace was used to perform continuous isothermal oxidation tests at 850°C, 950°C, and 1050°C, for 5 h, 12 h, 20 h, 40 h, 70 h, 100 h, and 140 h. Cover each crucible with a pot immediately after taking it out to avoid errors that might cause by the oxide film falling out during the cooling process. Each crucible and specimen group was weighed after air cooling, and the weight changes and oxidation rates were calculated to plot the oxidation kinetics curves. The composition of the oxide film was analyzed by X-ray diffraction (XRD, D-MAXIIA), with a scanning range of 20°-100°, with a Cu K_a_ radiation source and parameters of 40 kV and 40 mA. The surface and cross-section morphology and elemental composition of the oxide scale were analyzed by scanning electron microscopy (SEM, Gemini supra40) equipped with energy-dispersive X-ray spectroscopy (EDS).

## 3. Results and Discussion

### 3.1. Microstructure of Specimens

**Figure 1:**
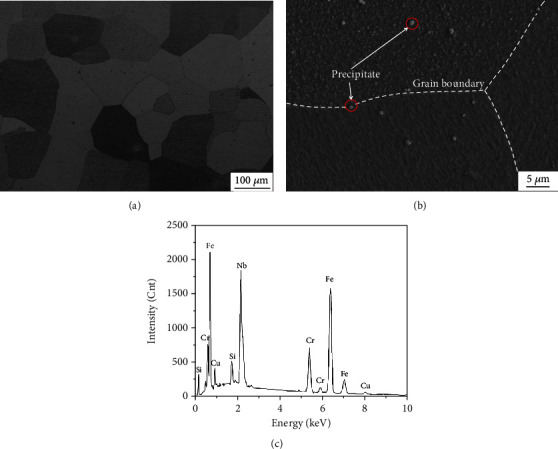
Microstructures of the annealed 17Cr-0.85Si-0.5Nb-1.2Cu ferritic stainless steel: (a) optical image of microstructure; (b) SEM image of microstructure; (c) corresponding EDS result of the precipitates in (b).

An optical micrograph of the steel after annealing at 1100°C for 30 min is shown in Figure 1(a), which reveals a typical equiaxed ferritic structure. The average grain size was measured to be 85 ± 5 *μ*m, and a small number of precipitated particles were observed in the grains and at the grain boundaries (Figure 1(b)). These precipitated particles were analyzed by EDS, and the results are shown in Figure 1(c). It indicates that these precipitates were residual Nb-containing compounds which have been reported by many references [16–21].

### 3.2. Oxidation Kinetics

**Figure 2:**
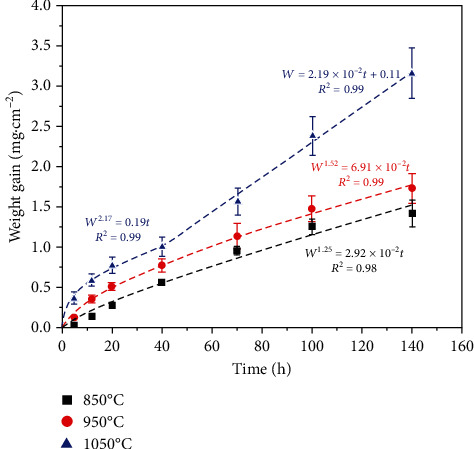
The oxidation weight gain curves of 17Cr-0.85Si-0.5Nb-1.2Cu ferritic heat-resistant stainless steel during exposure at temperatures between 850°C and 1050°C in air.

Figure 2 shows the oxidation weight gain curves of the studied steel at different temperatures. The weight gain gradually increased as the test temperature increased. At 850°C and 950°C, the weight gain curves followed a parabolic law, which indicated that the oxidation reaction was mainly controlled by elemental diffusion [22, 23]. Generally, Equation (1) can be used to describe the parabolic oxidation kinetics and is defined as [24]:

(1) $$\begin{array}{c} W^{2}=kt, \end{array}$$

where *W* is the oxidation weight gain in mg cm^−2^, *k* is the oxidation rate constant in mg^2^ cm^−4^ s^−1^, and *t* is the oxidation time in s. As we all know, the premise of the parabolic law is that there are no defects in the oxide film or at the oxide/matrix interface. However, after long-term oxidation during actual oxidation tests, cracks and other defects may form in the oxide scale, which will cause the oxidation kinetics law to deviate from the classic parabolic law. Therefore, the formula above can be modified as [16, 20]:

(2) $$\begin{array}{c} W^{n}=k_{\text{p}}t, \end{array}$$

where *n* is the index of oxidation and the value of *k*_p_ is determined by the mass gain per unit area and oxidation time. Generally, a larger value of *k*_p_ indicates a higher growth rate of oxidation film [25–27]. The oxidation kinetics equations of the experimental steel at 850°C and 950°C obtained from Equation (2) are:

(3) $$\begin{array}{c} W^{1.25}=2.92\times 10^{-2}t\left(850^{{}^{\circ}}\text{C}\right), \end{array}$$

(4) $$\begin{array}{c} W^{1.52}=6.91\times 10^{‐2}t\left(950^{{}^{\circ}}\text{C}\right). \end{array}$$

In these two equations, *n* < 2 indicates the formation of a loose oxide film, while the thickness of the oxide film did not increase proportionally to the element diffusion [28]. However, the oxidation still followed a parabolic law, indicating that the oxide film was protective at this time. Since *k*_p950°C_ > *k*_p850°C_, the oxidation resistance of the steel at 850°C was better than at 950°C, and the average oxidation rates at these two temperatures were 1.17 × 10^−2^ mg cm^−2^ h^−1^ and 2.05 × 10^−2^ mg cm^−2^ h^−1^, respectively.

At 1050°C, the oxidation weight gain curve of the studied steel was segmented, and the curve displayed a parabolic shape in the first 40 h and then became linear from 40 h to 140 h, which shows that the oxidation resistance of the steel decreased rapidly. The oxidation kinetics process can be expressed by Equation (5):

(5) $$\begin{array}{c} \left\{\begin{array}{c} W^{2.17}=0.19t,0\leq t\leq 40,\\ W=2.19\times 10^{-2}t+0.11,40\leq t\leq 140. \end{array}\right. \end{array}$$

When the oxidation time was less than 40 h at 1050°C, the *k*_p_ value was 0.19, which is 2.7 and 6.5 times higher than those obtained at 850°C and 950°C, respectively. Although the oxide film was still protective at this time, the overall oxidation resistance was significantly lowered compared to that at lower temperatures. When the oxidation time exceeded 40 h, the oxidation kinetics obeyed a linear law, and the oxidation rate was no longer controlled by diffusion processes in the oxide film but instead depended on the chemical reaction. At this stage, severe macroscopic peeling of the oxide film occurred, and the matrix was not completely covered, which sharply decreased the oxidation resistance of the steel [23, 29]. When the oxidation time reached 140 h at 1050°C, the weight gain was 3.16 mg/cm^2^, and the average oxidation rate was 3.66 × 10^−2^ mg cm^−2^ h^−1^.

### 3.3. Characterization and Analysis of Oxide Films

#### 3.3.1. Morphology and Surface Composition of Oxide Films

**Figure 3:**
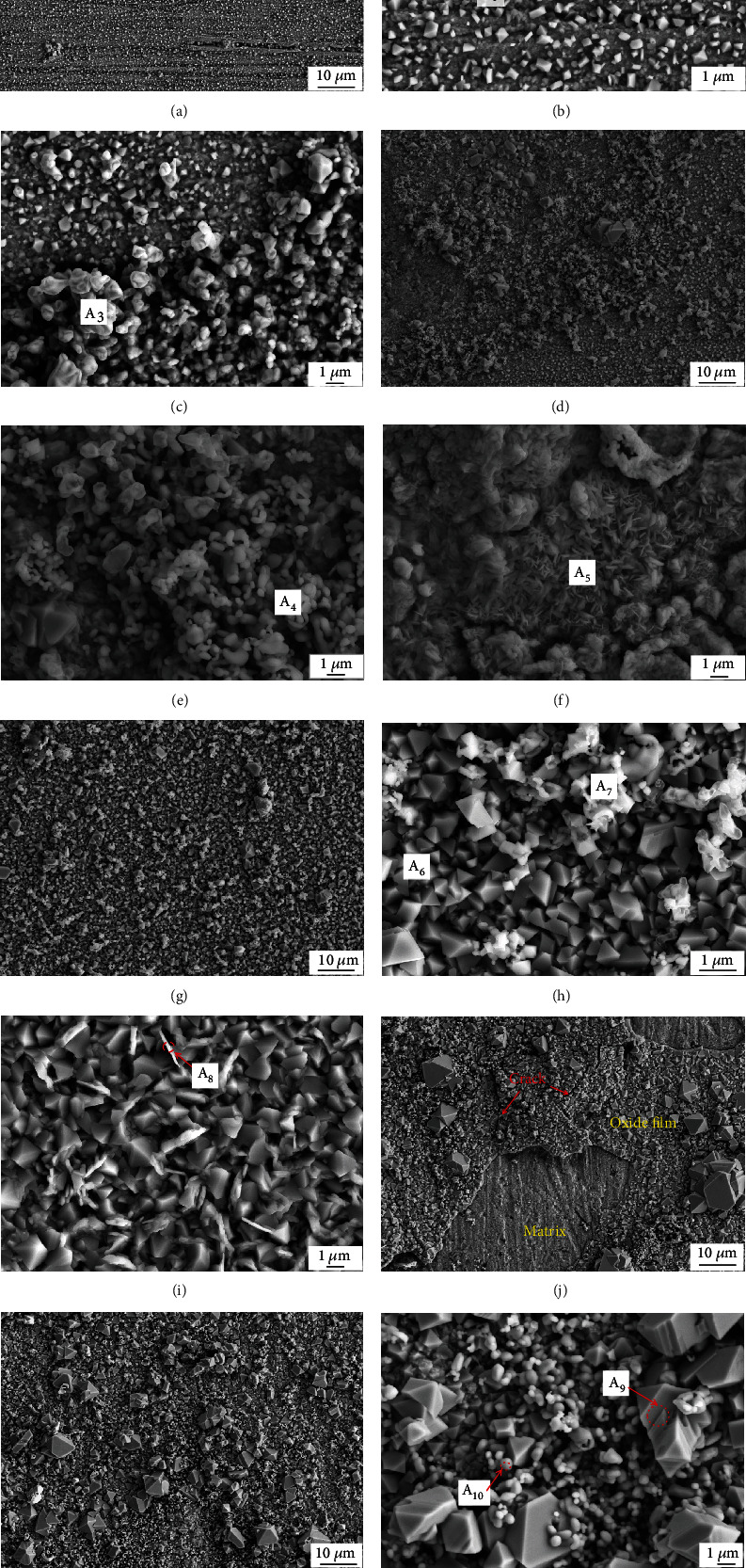
Surface morphology of oxide films at 850°C for (a–c) 5 h, (d–f) 20 h, (g–i) 70 h, and (j–l) 140 h in air.

The surface morphology of the studied steel after oxidation at 850°C for different times is shown in Figure 3, and the compositions of these oxides were further analyzed by EDS (Table 2). In the first 5 h (Figure 3(a)), surface scratches were apparent, indicating that the oxide film was very thin and the oxidation had just begun. At this time, the oxides were small cubes and bubble-like shapes (Figures 3(b) and 3(c)). The cubic oxides (A_1_) contained Cr, O, and a small amount of Cu, region A_2_ contained Cr, O, Fe, and Si, and the bubble-like particles in region A_4_ were identified as Cr_2_O_3_. No scratches were observed on the surface after 20 h oxidation (Figure 3(d)), and the size and quantity of the bubbly oxides on the surface increased and widely covered the thin cubic oxide film. In some areas, the bubble-like Cr_2_O_3_ oxides gradually merged (A_4_ in Figure 3(e)). Sparse large agglomerates and flocculent oxides (A_5_ in Figure 3(f)) which contained Cr, Fe, and Cu were also observed. The oxide film was even and compact after 70 h oxidation at 850°C, and a dense spinel oxide layer formed (Figures 3(g) and 3(h)). In addition to the cubic and a low amount of bubbly oxides, thin needle-like oxides (A_8_ in Figure 3(i)) also appeared. The cubic oxide films in these areas showed signs of fusion, and their geometric characteristics were not obvious. In Figure 3(h), two different oxides, A_6_ and A_7_, contained Cr, O, and Mn, and the cubic oxides (A_6_) contained more Mn, indicating that they were a spinel phase that contained Cr and Mn [30]. Area A_8_ mainly consisted of Cr, O, and a small quantity of Fe, Mn, and Cu. When the oxidation time was lengthened to 140 h (Figures 3(j) and 3(k)), the oxide film became rougher, and some areas collapsed after cooling. Many large spinel particles were produced (Figure 3(l)), with many granular oxides (A_10_) between them which were composed of Cr_2_O_3_, while the large layered cubic spinel oxides (A_9_) were mainly composed of Cr and Cu.

**Figure 4:**
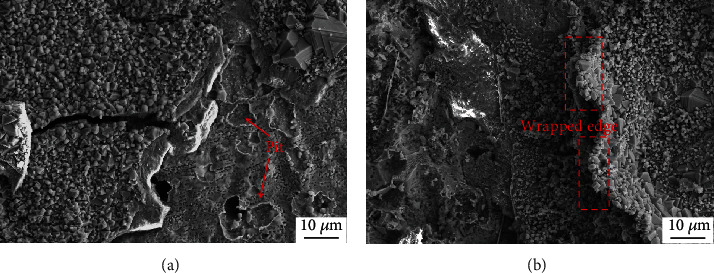
Surface morphology of oxide films formed after oxidation for 140 h in air at (a) 950°C and (b) 1050°C.

Figure 4 shows the surface morphology of the studied steel at 950°C and 1050°C after oxidation for 140 h in air. The surface oxide scales fell off to some degree when the time progressed to 140 h at both temperatures. It can be seen from Figure 4(a) that when the temperature was 950°C, the thickness of oxide scale was increased, and a pit-like morphology (mainly composed of Fe, Cr, Si, and O by EDS analysis) appeared in the area which experienced oxide film spallation. After oxidation at 1050°C, significant scale spalling was observed (Figure 4(b)). The remaining part of the oxide had a warped edge and was poorly attached to the substrate, indicating a significantly lower oxidation resistance. EDS results showed that the composition of the exfoliated area was oxides containing Mn, Cr, and Cu.

#### 3.3.2. Phase Analysis of Oxide Films

**Figure 5:**
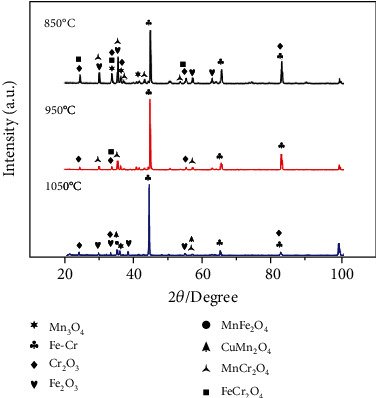
X-ray diffraction patterns of the oxide film surface after exposure at different temperatures for 140 h in air.

Figure 5 presents the XRD patterns of the oxide films obtained at different temperatures for 140 h. The oxidation products were similar and were mainly composed of Fe-Cr, Cr_2_O_3_, Fe_2_O_3_, Mn_3_O_4_, MnCr_2_O_4_, and FeCr_2_O_4_. But the intensities were different, indicating different phase content and/or thickness. The peaks of spinel Cu-Cr were not detected in the patterns, because their content was too low to be detected. At 1050°C, CuMn_2_O_4_ and more Fe_2_O_3_ appeared, compared to that at lower temperatures. The above results are both consistent with the EDS analysis. In addition, it can be found that as the temperature increased, the Cr_2_O_3_ content continued to decrease due to the further oxidation of Cr_2_O_3_ at high temperatures, which resulted in the formation of volatile CrO_3_. According to a previous report [31], this reaction proceeds very rapidly above 950°C, which is why Cr_2_O_3_ showed a low peak intensity at 1050°C. The XRD patterns also showed that the Fe-Cr peaks were heightened at higher temperatures, which could be due to severe peeling of the oxide film, which exposed more of the matrix.

#### 3.3.3. The Cross-Sectional Morphology of Oxide Scales

**Figure 6:**
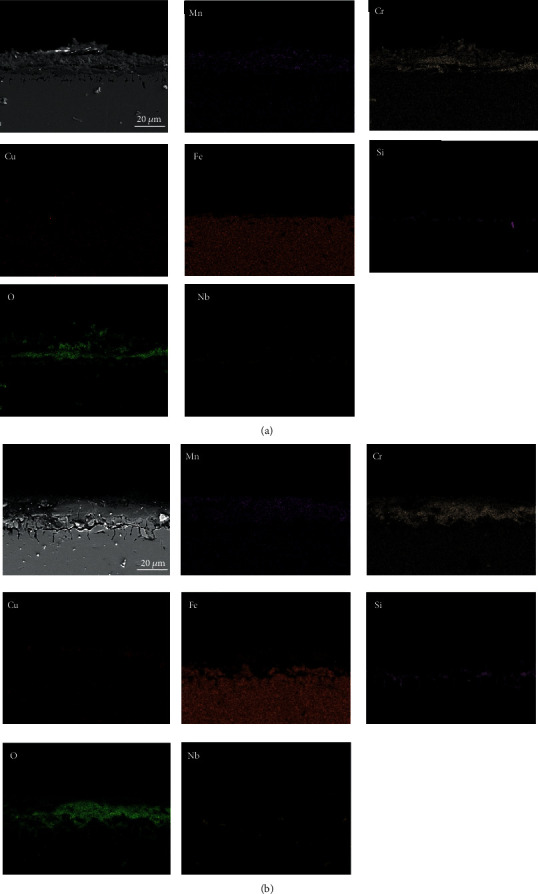
Cross-sectional energy spectral analysis of oxide films formed during exposure at 950°C in air: (a) for 12 h; (b) for 100 h.

**Figure 7:**
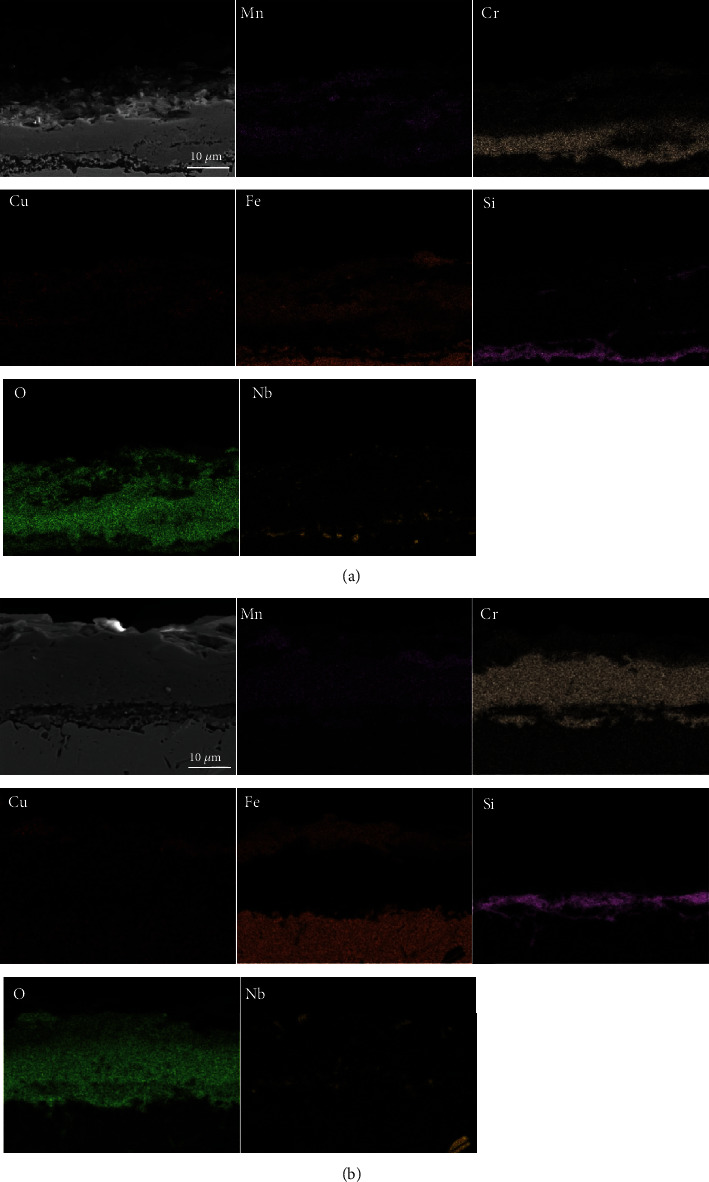
Cross-sectional energy spectral analysis of oxide films formed during exposure at 1050°C in air: (a) for 12 h; (b) for 100 h.

The cross-sectional morphology of the studied steel oxidized at 950°C and 1050°C for different times is shown in Figures 6 and 7, respectively. The changes in the distribution and concentration of O, Cr, Fe, Cu, Si, Nb, and Mn were detected. It can be seen from the figures that the oxygen content increased from the surface of the oxide film to the alloy substrate, and this indicated that the film at the interface of the substrate was denser than the surface oxide [1].

It can be seen from Figure 6(a) that when oxidized at 950°C for 12 h, the oxide film was still in the preliminary formation stage with a thickness of about 13.74 *μ*m. Mn was evenly distributed throughout the entire oxide layer, but the Cr content was greater at the oxide/steel interface. A tiny amount of Cu was observed in the outer oxide layer, while nearly no Fe and Nb were detected. Moreover, some Si-rich oxides distributed sporadically near the scale/steel interface. When the oxidation time was prolonged to 100 h (Figure 6(b)), the thickness of the oxide film increased to 19.28 *μ*m. Meanwhile, Fe began to gather near the oxide/metal interface, but Fe-rich oxides were not formed continuously or densely. A small amount of Cu was observed near the outermost side of the oxide scale, which may be related to the formation of Cu-Cr spinel oxide.

The cross-sectional element distribution of the oxide films after different oxidation times at 1050°C is shown in Figure 7. As the temperature increased, the oxide film gradually thickened and reached 18.75 *μ*m after 12 h (Figure 7(a)), and the elemental distribution was significantly different from the specimen exposed at 950°C. Si-rich oxides were distributed near the matrix continuously, while some Nb accumulation was scattered around them. A Fe-rich oxide layer was formed above the original Cr-rich scale, and the Cr-rich layer was thinner compared with that at 950°C. The concentration of Fe throughout the entire oxide film was significantly higher than that at 950°C, indicating that Fe diffused through the oxide film and reacted with oxygen. Mn was distributed evenly throughout the oxide film, and its concentration decreased further away from the outer layer. When the exposure time reached 100 h (Figure 7(b)), the outermost layer was a thick Fe-rich oxide, and the layer underneath was a Cr-rich oxide which was thicker than the one formed at 12 h. Large cavities also appeared in the inner Cr-rich layer, which might have resulted in the outward diffusion and oxidation of Fe at the scale surface. Therefore, it can be inferred that after being oxidized at 1050°C for 100 h, the oxide film was stratiform, and the outer layer of the scale was composed of Fe_2_O_3_ and a tiny amount of discrete CuMn_2_O_4_. The middle layer was composed of Cr_2_O_3_ together with Mn-rich spinel oxides, and the innermost layer was SiO_2_.

### 3.4. Oxidation Mechanism

#### 3.4.1. Oxidation Mechanism at 850°C and 950°C

According to thermodynamic theory, the affinity between oxygen and other elements in an alloy follows the order of Si > Nb > Mn > Cr > Fe > Cu [2, 25, 28, 32]. During the initial oxidation stage, Si and Nb are supposed to be first oxidized at the oxide/steel interface due to their higher diffusion coefficient and oxygen affinity in steel. However, there was a strong competition between Si and Nb for the interfacial oxidation [32]. Also, since the supply amount of Si atoms was much greater than that of Nb, NbO_2_ was difficult to form. This result corresponds to the result of X-ray diffraction, that is, no NbO_2_ was detected. The formed SiO_2_ layer was thin, porous, and discontinuous at this temperature; therefore, Cr and Mn began to diffuse outward through the SiO_2_ layer to generate Cr_2_O_3_ and MnO, respectively. As the oxidation time increased, a denser and more continuous protective Cr_2_O_3_ layer was formed, while Fe began to diffuse from the metal to the middle oxide layer to form FeO. However, the protective Cr_2_O_3_ layer acted as a barrier to Fe diffusion to the surface of the oxide film, and FeO and MnO reacted with Cr_2_O_3_, respectively, under the Cr_2_O_3_ layer and at the outermost oxide film surface to generate spinel oxides through the following reactions:

(6) $$\begin{array}{c} \text{MnO}\left(\text{s}\right)+\text{C}{\text{r}}_{2}{\text{O}}_{3}\left(\text{s}\right)\to \text{MnC}{\text{r}}_{2}{\text{O}}_{4}\left(\text{s}\right), \end{array}$$

(7) $$\begin{array}{c} \text{FeO}\left(\text{s}\right)+\text{C}{\text{r}}_{2}{\text{O}}_{3}\left(\text{s}\right)\to \text{FeC}{\text{r}}_{2}{\text{O}}_{4}\left(\text{s}\right). \end{array}$$

In addition, a small amount of Cu migrated to the out layer of the oxide scale and reacted with Cr to form spinel oxides after longer exposure times. These spinel oxides formed on the surface were a compact barrier for the inward diffusion of O, reducing the growth of the oxide scale and acting as protective layers against oxidation [23, 33, 34].

#### 3.4.2. Oxidation Mechanism at 1050°C

The oxidation rate of the studied steel increased significantly with increasing temperature. Si and Nb were oxidized during the initial oxidation period at 1050°C. And there are some differences from the low-temperature oxidation at 850°C −950°C. First of all, some sporadic Nb accumulation could be found. This is consistent with the finding of previous study, that is, when the content of Si in the steel is more than 0.5 wt.%, no Nb oxide aggregation layer will be formed [35]. Ali-Löytty et al. [16] also believed that the Nb-rich intermetallic precipitates remove free Nb from the alloy solution and thus made it difficult to form Nb-rich oxide layer. Therefore, based on the previous research conclusions and the phenomena observed in this experiment, we speculate that when the Si content is 0.85 wt.%, the oxidation of Nb has been severely suppressed, so the influence of Nb oxides will not be discussed here. Secondly, Si formed a continuous and dense thin oxide layer at the oxide/metal interface. It is known that Si-rich oxides are stable and protective at high temperatures [27, 36], yet a previous study [37] also found that it does not provide the highest oxidation resistance when a continuous Si oxide layer is formed at the steel/chromium interface. In contrast, it is better to obtain good oxidation resistance when a discontinuous Si oxide layer is formed at and below the interface with silica particles because Si does not act as a barrier for Cr diffusion to the chromium layer but rather prevents the diffusion of Cr from the matrix to the interface. Cr_2_O_3_ is also unstable at high temperatures, especially above 950°C, and easily reacts with oxygen to form volatile CrO_3_, which leads to the depletion of protective chromium oxides via the following reaction [25, 31, 38, 39]:

(8) $$\begin{array}{c} 2\text{C}{\text{r}}_{2}{\text{O}}_{3}\left(\text{s}\right)+3{\text{O}}_{2}\left(\text{g}\right)\to 4\text{Cr}{\text{O}}_{3}\left(\text{g}\right). \end{array}$$

When the average Cr content of the steel was lower than the critical concentration, the matrix Fe began to oxidize. Nodular oxides formed when Fe began to diffuse along the oxide layers and oxidized outward, which made the Cr_2_O_3_ film unstable, thereby causing more severe oxidation [40]. From Figure 7, it can be seen that Fe rapidly diffused through the inner scale, and oxidation occurred in the upper and middle oxide layers which were originally Cr_2_O_3_. Once the Fe-rich oxides which had faster oxidation rate began to form, no more dense protective chromium-rich oxide scale will be formed on the outermost surface. However, the Fe-rich oxide layer was loose and not protective, which provided more paths for the diffusion of Fe, O, Cr, and Mn [31]. It can also be observed in Figure 7(a) that the Fe-rich oxide had a bilayer structure whose outer layer was Fe_2_O_3_, and the inner was Fe-Cr oxides, but they were not dense enough to impede the outward diffusion of ions. Moreover, due to the different Pilling-Bedworth ratios of these oxides, cracks also formed due to growth stress [41, 42]. The above reasons eventually led to the failure of the oxide scale at 1050°C. Some Cu-rich and Mn-rich oxides were also distributed on the outermost side of the oxide film when the exposure time was extended to 100 h. It was estimated that few CuMn_2_O_4_ and MnFe_2_O_4_ spinel oxides were formed. Previous studies have shown that when a Cu-rich phase segregates at the scale-matrix interface, it can hinder the inward diffusion of oxygen, thereby improving the oxidation resistance [43], but this phenomenon was not observed in this study. In addition, another research showed that Cu-Mn spinel oxides are good barrier to the diffusion of Cr [44–46]. However, since the nonprotective Fe_2_O_3_ dominated the outermost film, spinel oxides did not form a strong protective continuous dense layer, and the oxidation rate of the experimental steel increased linearly.

## 4. Conclusions

The isothermal oxidation behavior of 17Cr-0.85Si-0.5Nb-1.2Cu ferritic stainless steel at 850°C, 950°C, and 1050°C in air was studied, and the following conclusions were obtained:

1. The oxidation rate increased with the increasing temperature. The oxidation kinetics curves conformed to the parabolic law at 850°C and 950°C, while the kinetics curve followed a parabolic-linear rule at 1050°C
2. At 850°C and 950°C, a small amount of oxide film peeled off. The oxide film was composed of a small amount of SiO_2_ and continuous and dense Cr_2_O_3_, FeCr_2_O_4_, and Cr-Mn spinel oxides. The composition of the oxide film was sufficiently stable to protect the matrix from further acute oxidation
3. The spallation of the oxide scale became more severe when the exposure time increased to 40 h at 1050°C. The severe volatilization of Cr_2_O_3_ and the enrichment of nonprotective Fe-rich oxides on the surface rapidly decrease the oxidation resistance at this high temperature

## Figures and Tables

**Table 1:** Chemical composition of the experimental ferritic stainless steel (wt.%).

C	Si	Mn	Cr	Nb	Cu	P	S	Fe
0.01	0.85	0.25	17.14	0.5	1.2	0.008	0.004	Bal.

**Table 2:** EDS quantitative analysis results of the different regions in Figure 3.

Test point	Chemical composition (at.%)					
	O	Fe	Cr	Mn	Cu	Si
A1	65.63	—	31.25	—	3.12	—
A2	39.45	9.09	49.94	—	—	1.52
A3	57.98	—	42.02	—	—	—
A4	47.86	—	52.14	—	—	—
A5	57.13	16.67	20.97	—	5.22	—
A6	34.17	—	42.77	23.06	—	—
A7	43.47	—	43.87	12.66	—	—
A8	50.42	8.72	30.96	6.44	3.46	—
A9	57.22	—	39.20	—	3.58	—
A10	52.55	—	47.45	—	—	—

## Data Availability

The raw/processed data required to reproduce these findings cannot be shared at this time as the data also forms part of an ongoing study.
